# Stevens’ Cure (Umckaloabo)—the vindication of a patent medicine

**DOI:** 10.3389/fphar.2023.1294997

**Published:** 2024-01-03

**Authors:** T. Brendler, M. A. Stander, B.-E. van Wyk

**Affiliations:** ^1^ Department of Botany and Plant Biotechnology, University of Johannesburg, Johannesburg, South Africa; ^2^ Department of Biochemistry, University of Stellenbosch, Stellenbosch, South Africa; ^3^ Mass Spectrometry Unit, Central Analytical Facility, University of Stellenbosch, Stellenbosch, South Africa

**Keywords:** Umckaloabo, Stevens’ Cure, *Pelargonium sidoides* DC., *Pelargonium reniforme* Curt., umckalin, identification

## Abstract

Stevens’ Cure (Umckaloabo) emerged as a patent medicine claiming to treat tuberculosis in the United Kingdom at the beginning of the 20th century. However, due to its identity being shrouded in secrecy, it was never truly accepted by the medical community. It was “rediscovered” in the 1970s and subsequently developed into a very popular and successful phytopharmaceutical for the treatment of upper respiratory tract infections. Whether Stevens’ Cure contained the same ingredient(s) as the modern Umckaloabo has not yet been demonstrated. We attempted to elucidate for the first time the identity of the original ingredient by comparative analysis of historical product samples. Three historical samples of Stevens’ Cure were compared with *Pelargonium sidoides* DC. and *P. reniforme* Curt. root per UPLC-MS analysis. We confirm that the ingredient–*P. sidoides* DC.—is indeed the same as used in modern phytotherapy. We also attribute the first ethnopharmacological record of *P. sidoides* DC. being used for the treatment of tuberculosis to C. H. Stevens, the “creator” of Umckaloabo.

## 1 Introduction

Stevens’ Cure, today better known as Umckaloabo, was introduced to the United Kingdom as a patent medicine at the end of the 19th century. Similar to other early introductions of southern African medicinal herbs, such as devil’s claw (*Harpagophytum* spp.), rooibos [*Aspalathus linearis* (Burm.f.) R.Dahlgren], honeybush (*Cyclopia* spp.), buchu (*Agathosma* spp.), Cape aloe (*Aloe ferox* Mill.) and uzara [*Xysmalobium undulatum* (L.) W.T. Aiton] (Stander et al., 2019; Brendler, 2021; Brendler and Abdel-Tawab, 2022; Brendler and Cock, 2022; Brendler et al., 2023), its arrival in Europe was driven by entrepreneurship and, as opposed to later (and less successful) attempts, such as hoodia [*Hoodia gordonii* (Masson) Sweet ex Decne] and sceletium (*Mesembryanthemum tortuosum* L.) (Brendler, 2020; Brendler et al., 2021), uninhibited by regulations. Suffering from tuberculosis, Charles H. Stevens was sent by his doctor for convalescence to South Africa, where he was miraculously cured by a local Sotho healer. He brought the cure, an unidentified botanical, back to England and began to market it to fellow sufferers as a patent medicine branded as Umckaloabo. Aggressive advertising (by mail, in dailies, through anonymously published books and paraphernalia such as bookmarks) made his business flourish. Stevens was open to the medical establishment, promoting his remedy on multiple occasions, however, his secrecy and claims of a cure for tuberculosis did not pair well with the scrutiny of science and he was largely ignored or labelled a quack (British Medical Association, 1909a; British Medical Association, 1909b; American Medical Association, 1910; American Medical Association, 1930). It is Stevens’ contentious nature that we owe the opportunity to–belatedly–uncover his secret. He pressed multiple litigations against the medical establishment (British Medical Association, 1912) but lost due to his unwillingness to disclose the identity of his ingredient, among other reasons. However, every one of those court cases led to attempts to elucidate the composition of his remedy: samples were procured, government officials tasked, and renowned botanical institutes involved (Misc, 1936ff.). Even though none of these attempts were successful, they left a paper trail and the samples ended up in official collections. We were able to locate four samples in the Economic Botany Collection of the Royal Botanical Gardens, Kew, three of which could be authenticated as Stevens’ Cure beyond a reasonable doubt (see Section 2.1). These were analyzed and results are presented below. Stevens’ success also caught international attention, and the Swiss French physician Adrien Sechehaye started to treat his patients with Umckaloabo and reported his successes to the European medical societies (Sechehaye, 1923; Sechehaye, 1934). Soon, interest was raised also in Germany (Bojanowski, 1937). That may be the reason for why after Stevens’ death the “brand” and its secret ended up there. In parallel, however, antibiotics became available for the treatment of tuberculosis, and Umckaloabo fell to the wayside until it was revived by the quest of German scientist Sabine Bladt in the early 1970s (Bladt, 1974). Bladt, curiously, was unaware of the historic samples, or at least nothing in her published work hints at her knowing of their existence. Her ethnobotanical and biochemical approach led to the identification of *Pelargonium reniforme* as the source plant of Umckaloabo (Bladt, 1977). In retrospect, it must be assumed that her collected plant material was not correctly identified (pers. comm. K. P. Latté, July 2020), since she reported the presence of umckalin, which was subsequently found to be the marker compound for *P. sidoides* (Kayser, 1997), while absent in *P. reniforme* (Latté, 1999). In fact, Kolodziej and co-workers (Kolodziej, 2000; Kolodziej et al., 2002; Kolodziej, 2007) have identified several compounds including coumarin and coumarin sulfates (umckalin and umckalin sulfate) in *P. sidoides* that were not detected in *P. reniforme*. The presence of umckalin as a unique chemical marker for *P. sidoides* was also confirmed by Viljoen and colleagues (Viljoen et al., 2015).

The transformation of Umckaloabo from a patent medicine into a modern phytopharmaceutical has been reviewed by us previously (Brendler and van Wyk, 2008; Brendler, 2009). Since then, more than 100 publications have been added to the already impressive body of data on biochemistry, pharmacology, clinical efficacy, and safety of Umckaloabo. The contemporary brand owner (Schwabe Group, Karlsruhe, Germany) has promoted the investigation of Umckaloabo (EPs^®^ 7630^1^) resulting in more than 30 clinical trials over the last 25 years (total study population >10,000) for the treatment of acute respiratory tract infections. A Cochrane review of eight studies (Timmer et al., 2013) found some evidence for efficacy but deemed the overall quality low. More recent reviews (Matthys et al., 2016; Careddu and Pettenazzo, 2018; Seifert et al., 2019) included a larger body of data in their reviews and meta-analyses, and attested efficacy in children, adolescents and adult patients with acute bronchitis, rhinosinusitis or tonsillopharyngitis. An investigation of EPs^®^ 7630 effect on respiratory viruses (Michaelis et al., 2011) and its excellent safety profile (Kamin et al., 2018; Schapowal et al., 2019) led to it being discussed as having potential to affect the human immune response in the context of COVID-19 (Brendler et al., 2020), which has since been confirmed *in vitro* and *in vivo* (Papies et al., 2021; Emanuel et al., 2023).

## 2 Materials and methods

### 2.1 Samples and sampling

Commercial samples of dried *Pelargonium sidoides* and *P. reniforme* roots were supplied by Ulrich Feiter of Parceval (Pty) Ltd., Wellington, South Africa. The batch numbers were 20090, 20091, and 20092 for *P. sidoides,* and 2023, 20094, and 20095 for *P. reniforme*. The three historic samples, EBC 45821, EBC 45819 and EBC 77377 were procured from the Economic Botany Collection of the Royal Botanic Gardens, Kew, United Kingdom. The authenticity of those samples could be confirmed with the help of catalogue notes and by relating them to a file labelled “Umckaloaba” [sic!] of various correspondence regarding the British Medical Association’s attempts to identify the active ingredient in Stevens’ Cure (Misc, 1936ff.) (EBS 45821 and EBS 77377), whereas EBS 45819 was contained in an envelope addressed to the Royal Botanic Gardens from Pharmacie Hahn, Geneva, 1922. Adrien Sechehaye mentioned this pharmacy as his supplier for Umckaloabo (Helmstädter, 1996).

### 2.2 Extraction

Approximately 0.2 g of dry plant material was extracted with 50% methanol in water containing 1% formic acid (2 mL) in a 15 mL polypropylene centrifuge tube by soaking it overnight, followed by extraction in an ultrasonic bath (0.5 Hz, Integral systems, RSA) for 60 min at room temperature. The extracts were centrifuged (Hermle Z160m, 3,000 × g for 5 min) to remove any particulates and transferred into vials. An additional analysis was performed on the hydrolyzed samples.

### 2.3 Standards

Standards of umckalin, gallic acid and citric acid were obtained from Sigma-Aldrich. Standard solutions of umckalin were prepared quantitatively ranging from 1 to 100 μg/mL in concentration in methanol.

### 2.4 LC-MS analysis

A Waters Synapt G2 Quadrupole time-of-flight (QTOF) mass spectrometer (MS) connected to a Waters Acquity ultra performance liquid chromatograph (UPLC) (Waters, Milford, MA, United States) was used for high resolution UPLC-MS analysis. In short electrospray ionization in the negative mode was used, a Waters HSS T3, 2.1 mm × 100 mm, 1.7 μm column and mobile phase gradient of 0.1% formic acid (Solvent A) and acetonitrile containing 0.1% formic acid as solvent B (Stander et al., 2017). The flow rate was 0.25 mL/min, the gradient started with a 1-min hold at 100% (A) followed by a linear gradient to 28% solvent (B) in 22 min and another linear gradient over 50 s to 40%, a wash step at 100% (B) for a minute and re-equilibration to initial conditions over 4 min to give a total run time of 29 min. The column was at 60°C. Data was acquired in MS^E^ mode where both low energy data and high energy fragmentation data are acquired.

## 3 Results

**FIGURE 1:**
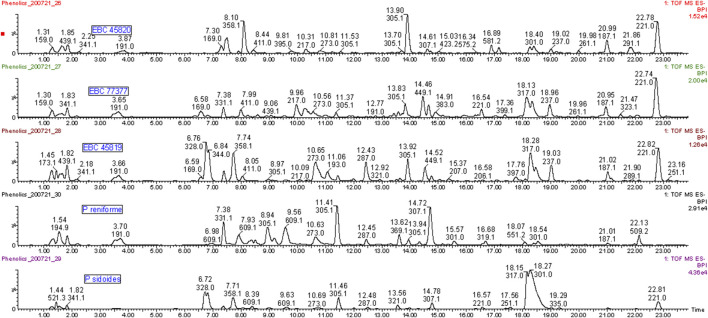
Phenolic compounds in extracts from authentic root samples of *Pelargonium reniforme, P. sidoides* and three historical samples of Stevens’ Cure (EBC 45819, EBC 77377, EBC 45820). Umckalin (Rt ∼22.8 min) is a known marker compound for *P. sidoides*. Umckalin sulfate (Rt ∼18.3 min) occurs at high levels in *P. sidoides* and the Kew samples but not in *P. reniforme*. Epigallocatechin (Rt 11.4 min) and gallocatechin (Rt 8.9 min) are major compounds in *P. reniforme* and only minor compounds in *P. sidoides* and the Kew samples. The turgorins (m/z 328 and 358, 6.7 and 8.09 min) are present in the historical samples and *P. sidoides* and not in *P. reniforme*.

Direct evidence of the botanical identity of the three historical samples of Stevens’ Cure is revealed here for the first time (Figure 1).

Umckalin (*m/z* 221.0454, 22.8 min) is a known chemical marker for *P. sidoides* (Viljoen et al., 2015) and occurred as a major phenolic compound in all three of the Kew samples (363–738 mg/kg in the latter–see Figure 1; Supplementary Material).

**FIGURE 2:**
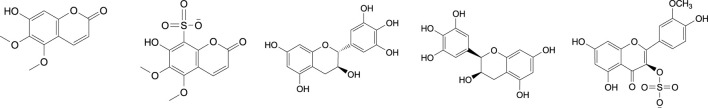
Structure of the main markers of *Pelargonium sidoides* (umckalin, umckalin sulfate, gallocatechin and epi-gallocatechin) and persicarin (isorhamnetin-3-sulfate), the first isolated sulfated flavonoid (left to right).

Umckalin sulfate (6-hydroxy-5,7-dimethylcoumarin-8-sulfate, *m/z* 301.0009, 17.45 min), is a major compound in *P. sidoides* (Kolodziej, 2000; Kolodziej et al., 2002; Kolodziej, 2007) and in two of the three museum samples and a minor compound in the third. Epigallocatechin (*m/z* 305.0664, 11.1 min) is a major compound in *P. reniforme* and only a minor one in *P. sidoides* and the three historical samples. Note, that there are more than 1 *m/z* 305 peaks in Figure 1—the one at 11.4 min is epigallocatechin, the one at 8.9 min (*P. reniforme* only) is gallocatechin and the one at 13 min an unidentified flavonoid sulfate. Sulfated flavonoids are an uncommon group of compounds but have been found in some plant families. Teles et al. (2018) recently published a review on these compounds. Persicarin (isorhamnetin-3-sulfate) is the first sulfated flavonoid reported, the sulfate is an O-sulfate, whereas with umckalin-sulfate it is a C-sulfate. O-sulfates forms a strong [HSO_4_^−^] *m/z* 96.96 fragment, whereas C-sulfates forms a [M-H-SO_3_]^−^ as a main fragment ion. See Figure 2 for the structures and Supplementary Material for the spectra and fragmentation information of the marker compounds. An overview of compounds tentatively identified in *Pelargonium* extracts in this study is provided in Table 1.

**TABLE 1:** List of compounds tentatively identified in *Pelargonium* extracts in this study showing, retention time, detected [M-H] ion, elemental composition and MS^E^ fragments (with the base peaks in bold) as well as literature references to where the compounds were previously detected.

Time	*m/z*	[M-H]^−^	MS^E^ fragment ions	Tentative identification	References
2.73	191.0196	C_6_H_7_O_7_	**111.0086**, 87.0093, 85.0305, 67.0197	Citric acid	Standard
6.54	169.0132	C_7_H_5_O_5_	125.0253	Gallic acid	Standard
6.77	328.044	C_10_H_11_N_5_O_6_P	134.0464	K-LMF 3	Schildknecht (1984)
6.84	344.0396	C_10_H_11_N_5_O_7_P	**150.0427**, 133.0148, 78.9575	K-LMF 2	Schildknecht (1984)
7.33	331.1025	C_14_H_19_O_9_	139.0399, **169.0504**	Koaburaside	Chung et al. (1997)
7.69	358.0538	C_11_H_13_N_5_O_7_P	**164.0568**, 133.0144, 78.9575	Methoxy LMF 2	**New**
7.84	411.0127	C_13_H_15_O_13_S	**241.0038**, 169.0153, 125.0251, 96.9602	PLMF 1	Schildknecht (1984)
7.93	609.1234	C_30_H_25_O_14_	441.0830, 423.0706, 305.0659, 272.9700, 193.0133, 177.0190, **125.0234**	(epi) Gallocatechin Dimer	Callemien and Collin (2008)
8.36	609.1243	C_30_H_25_O_14_	441.0830, 423.0690, 409.0430, 305.0659, 272.9694, 193.0133, 177.0190, **125.0244**	(epi) Gallocatechin Dimer	Callemien and Collin (2008)
8.77	305.065	C_15_H_13_O_7_	221.0451, 219.0695, 167.0366, 139.0405, 137.0251, **125.0242**, 111.0467, 109.0299	Gallocatechin	Kolodziej (2000)
9.24	217.0173	C_8_H_9_O_5_S	**96.9583**, 193.0120	Unknown flavonoid sulfate	**New**
9.64	913.1762	C_34_H_41_O_29_	609.1199, 441.0815, 423.0703, 305.0645, 177.0192, **125.0241**	(epi) Gallocatechin Trimer	Callemien and Collin (2008)
10.3	365.0172	C_12_H_13_O_11_S	347.0041, **210.9911**, 153.0182, 122.9763, 109.0287, 96.9592	Phenolic glycoside sulfate	Pereira et al. (2015)
10.65	272.9704	C_9_H_5_O_8_S	**193.0143**, 175.0061, 149.0240, 121.0299, 93.0346, 77.0383	Dihydroxy coumarin sulfate	Gödecke et al. (2005)
11.06	272.9798	C_9_H_5_O_8_S	**193.0137**, 177.0175	Dihydroxy coumarin sulfate	Gödecke et al. (2005)
11.4	305.0664	C_15_H_13_O_7_	219.0676, 179.0377, 167.0336, 151.0371, 139.0394, 137.0238, **125.0241**, 109.0293	Epigallocatechin	Kolodziej (2000)
11.99	485.1284	C_21_H_25_O_13_	303.0444, **177.0183**, 125.0233	Gallocatechin O-hexoside	New
12.77	191.0337	C_10_H_7_O_4_	125.0222	Scopoletin	Kayser and Kolodziej (1995)
12.43	286.9853	C_10_H_7_O_8_S	207.0294, **192.0061**, 164.0107, 108.0211	Hydroxy methoxy coumarin sulfate	**New**
13.8	305.0686	C_12_H_17_O_7_S	225.1135, 165.0938, 147.0842, **96.9598**, 59.0126	Unknown flavonoid sulfate	**New**
13.58	369.0815	C_16_H_17_O_10_	**192.0059**, 207.0292, 163.0040	Unknown	
14.34	289.0736	C_12_H_17_O_6_S	125.0246, 176.1043??	Epicatechin	Kolodziej (2000)
14.6	449.1058	C_21_H_21_O_11_	**287.0547**, 269.0450, 259.0606, 125.0240	Eriodictoyl-hexoside	**New**
14.7	307.085	C_12_H_17_O_7_S	227.1297, 167.1039, 123.0820, **96.9596**, 59.0128	Unknown flavonoid sulfate	**New**
15.5	301.0004	C_11_H_10_SO_8_	221.0437, 206.0199, **190.9985**, 163.0031, 135.0099, 125.0228	Fraxidin sulfate	Kolodziej et al. (2002)
16.53	221.0445	C_11_H_9_O_5_	**190.9990**, 163.0049, 91.0186	Fraxidin/fraxinol	Kayser and Kolodziej (1995)
16.56	319.0798	C_16_H_15_O_7_	179.0363, 164.0126, **139.0403**, 125.0236, 111.0448	Unknown	
16.8	581.2255	C_28_H_37_O_13_	419.1681, 404.1408, 373.1212, 227.1284, 153.0561	Unknown	
17.15	581.2236	C_28_H_37_O_13_	419.1750, 373.1436, 227.1278, 153.0562	Unknown	
18.2	316.9959	C_11_H_9_O_9_S	237.0402, 222.0173, **206.9928**, 178.9978, 151.0045, 107.0139, 95.0131, 67.0180	8-hydroxy-5,7 dimethoxycoumarin-6-sulfate	Kolodziej et al. (2002)
18.6	301.0009	C_11_H_10_SO_8_	221.0457, 206.0226, **190.9987**, 163.0037, 135.0087, 119.0145, 95.0134, 91.0190	5,6 dimethoxycoumarin-7-sulfate	Kolodziej et al. (2002)
19.03	237.0394	C_11_H_9_O_6_	222.0145, **206.9935**, 178.9966, 151.0046, 123.0092, 95.0135, 67.0182	6,8-dihydroxy-5,7-dimethoxycoumarin	Kayser and Kolodziej (1995)
21	187.0965	C_9_H_15_O_4_	Solvent contaminant		
22.1	509.2018	C_25_H_33_O_11_	289.1097, 177.0164	Unknown compound	
22.8	221.0454	C_11_H_9_O_5_	206.0216, **190.9977**, 163.0034, 135.0119, 119.0149, 95.0130, 91.0182	Umckalin (7-hydroxy-5,6-dimethoxycoumarin)	Kayser and Kolodziej (1995)
26.78	415.1773	C_20_H_31_O_7_S	**96.9601**, 79.9564	Unknown flavonoid sulfate	**New**
29	399.1832	C_20_H_31_O_6_S	**96.9597**, 79.9589	Unknown flavonoid sulfate	**New**
29.52	399.1823	C_20_H_31_O_6_S	**96.9585**	Unknown flavonoid sulfate	**New**

The coumarin sulfates can be distinguished from the flavonoid sulfates in the fragment ions. The coumarin sulfates have a base peak or strong fragment ion at [M-H-SO_3_]^−^ where the sulfate is on the coumarin ring whereas if the sulfate is on a flavonoid glycoside on another position, a fragment ion at *m/z* 96.96 is detected for [HSO_4_]^−^. It is challenging to identify the sulfated flavonoid glycosides, because only the elemental composition and the one fragment (*m/z* 96.96) is mostly detected and the compounds are not stable enough to isolate preparatively (Schötz, K., Nöldner, 2006).

Interestingly, sulfated flavonoids have been shown to have antimicrobial affects and they are also some of the main metabolites found in human blood after administration of the aglycones (Teles et al., 2018).

The turgorins (LMF 3, *m/z* 328.0440, 6.77; LMF 2, *m/z* 344.0596, 6.8 min) are present in the historical samples and *P. sidoides* but not in *P. reniforme* (Schildknecht, 1984).

## 4 Discussion

While the presence of a small amount of *P. reniforme* in the historical samples cannot be ruled out with certainty, it is safe to state that most of the plant material is from *P. sidoides*. The quantitative results are presented in the Supplementary Material. In early attempts to elucidate the composition of Stevens’ Cure it was speculated that it may belong to the genus *Rumex* (catalogue note to EBS 45819). However, no unique peaks corresponding to the presence of anthraquinones (chrysophanol and its glycosides) were detected in the samples that were not present in *P. sidoides* in both positive (data not shown) and negative mode.

Tuberculosis and/or respiratory ailments do not appear in the historical southern African literature prior to the 20th century (Brendler and van Wyk, 2008). The earliest record by Sanderson (ca. 1860) is from the Eastern Free State (adjoining Lesotho), where Khoi people were said to use the plant as a cure for unspecified ailments (Smith, 1966).

The earliest record from the Eastern Cape documented the species as *iYeza lezikhali* and *iKhubalo* in isiXhosa and reported it to be used for “dysentery, attended by inflammation and fever” (Smith, 1895). In Lesotho, *P. sidoides* was known as *khoaara e nyenyane* (in Sesotho) and the roots used to treat colic (Phillips, 1917). Stevens claimed that the plant was known as *Umckaloabo* in Basutoland (now Lesotho) and that the treatment prescribed by a Basotho healer completely cured him of his tuberculosis (Sechehaye, 1934). We now know that the given provenance of the plant, which included the Gold Coast and Liberia (Anonymous, 1931), and the botanical affinity given as Polygonaceae, were deliberate attempts at hiding the geographical origin and identity of the plant material. Nevertheless, the recorded use against tuberculosis is a valuable original ethnobotanical record for the species, that may have been completely forgotten had it not been for Stevens.

*Pelargonium sidoides* occurs neither in the Western Cape Province, nor in KwaZulu-Natal Province and Eswatini. The report by Watt and Breyer-Brandwijk (Watt and Breyer-Brandwijk, 1962) that the plant was used in the Cape as an “old remedy for delay in the onset of menses” was based on Kling (Kling, 1923), who merely cited the well-established history of this indication for *P. grossularioides* (L.) L’Herit (at the time known as *P. anceps* DC.). The confusion was due to the shared Malay/Afrikaans vernacular names (*rabas* or *rabassam*), first recorded by Pappe (Pappe, 1847; 1850) and later by Kling (1923) and Laidler (Laidler, 1928). Therefore, data in Watt and Breyer-Brandwijk (1962) were probably inaccurate but taken at face value by later authors (Hutchings et al., 1996). These authors added the treatment of severe diarrhea, a prolapsed rectum, severe gonorrhea, and a stomach ailment in babies known as *intisila* to the repertoire of the species, claimed to be used by the Zulu and Swazi.

## 5 Conclusion

The analysis of historical samples leaves no doubt that Stevens’ Cure was prepared from *Pelargonium sidoides*, a Sotho medicine traditionally used in Lesotho against colic and, according to Stevens (Sechehaye, 1934), also against tuberculosis. Recorded uses in South Africa do not include respiratory ailments until the late 20th century. Although Stevens’ claim of a cure for tuberculosis was never validated beyond pre-clinical investigations (Seidel and Taylor, 2004; Kim et al., 2009; Qasaymeh et al., 2019), the use of his remedy is vindicated, not only by its botanical identity but also by several clinical studies showing support for the treatment of respiratory infections.

## Data Availability

The original contributions presented in the study are included in the article/Supplementary Material, further inquiries can be directed to the corresponding author.

## Footnotes

1 Registered trademark of Dr. Willmar Schwabe GmbH & Co. KG, Karlsruhe, Germany

## References

[B1] American Medical Association (1910). Exposure of a quack. JAMA 55, 872. 10.1001/jama.1910.04330100061021

[B2] American Medical Association (1930). Stevens' consumption cure. Declared a fraud and debarred from the mails. JAMA 95, 951. 10.1001/jama.1930.02720130047027

[B3] Anonymous (1931). Tuberculosis - its treatment and cure with the help of Umckaloabo. London: B. Fraser & Co.

[B4] Bladt S. (1974). *Zur Chemie der Inhaltsstoffe der Pelargonium reniforme Curt. - wurzel (Umckaloabo).* PhD. Munich, Germany: Ludwig-Maximilians-Universität.

[B5] Bladt S. (1977). Umckaloabo - droge der afrikanischen Volksmedizin. Dtsch. Apoth. Ztg. 117, 1655–1660.

[B6] Bojanowski W. (1937). Das biologische tuberkulosemittel umckaloabo. Fortschr. Med. 55, 1.

[B7] Brendler T. (2021). From bush medicine to modern phytopharmaceutical: a bibliographic review of devil’s claw (harpagophytum spp). Pharmaceuticals 14, 726. 10.3390/ph14080726 34451822 PMC8398729

[B8] Brendler T. (2009). “Umckaloabo: from a patent remedy to a modern herbal pharmaceutical based on *Pelargonium sidoides* with clinically proven efficacy,” in African natural plant products I: new discoveries and challenges in chemistry and quality, ACS symposium series. Editor Juliani R. (Washington, DC: ACS Publications), 295–319.

[B9] Brendler T. (2020). “The rise and fall of *hoodia*: a lesson on the art and science of natural product commercialization,” in African natural plant products III: discoveries and innovations in chemistry, bioactivity, and applications, ACS symposium series. Editor Juliani R. (Washington, DC: ACS Publications), 313–324.

[B10] Brendler T. Van Wyk B.-E. (2008). A historical, scientific and commercial perspective on the medicinal use of Pelargonium sidoides (Geraniaceae). J. Ethnopharmacol. 119, 420–433. 10.1016/j.jep.2008.07.037 18725280

[B11] Brendler T. Abdel-Tawab M. (2022). Buchu (*Agathosma betulina* and *A. crenulata*): rightfully forgotten or underutilized? Front. Pharmacol. 13, 813142. 10.3389/fphar.2022.813142 35197854 PMC8859318

[B12] Brendler T. Al-Harrasi A. Bauer R. Gafner S. Hardy M. L. Heinrich M. (2020). Botanical drugs and supplements affecting the immune response in the time of COVID-19: implications for research and clinical practice. Phytother. Res. 35, 3013–3031. 10.1002/ptr.7008 33373071

[B13] Brendler T. Brinckmann J. A. Feiter U. Gericke N. Lang L. Pozharitskaya O. N. (2021). *Sceletium* for managing anxiety, depression and cognitive impairment: a traditional herbal medicine in modern-day regulatory systems. Curr. Neuropharm. 19, 1384–1400. 10.2174/1570159X19666210215124737 PMC8762184 33588735

[B14] Brendler T. Cameron S. Kuchta K. (2023). Uzara (Xysmalobium undulatum) - an underutilized anti-diarrhoeic and spasmolytic herbal remedy. J. Ethnopharmacol. 318, 116999. 10.1016/j.jep.2023.116999 37549862

[B15] Brendler T. Cock I. E. (2022). Cape aloe bitters – past and present. S. Afr. J. Bot. 147, 1016–1026. 10.1016/j.sajb.2021.11.054

[B19] British Medical Association (1909a). Secret remedies: what they cost and what they contain. London: British Medical Association.

[B20] British Medical Association (1909b). Stevens' consumption cure. BMJ, 672.

[B21] British Medical Association (1912). Stevens v. British medical association. BMJ 1250–1255, 1341–1344. 1170–1171.

[B22] Callemien D. Collin S. (2008). Use of RP-HPLC-ESI (–)-MS/MS to differentiate various proanthocyanidin isomers in lager beer extracts. J. Am. Soc. Brew. Chem. 66, 109–115. 10.1094/asbcj-2008-0215-01

[B23] Careddu D. Pettenazzo A. (2018). *Pelargonium sidoides* extract EPs 7630: a review of its clinical efficacy and safety for treating acute respiratory tract infections in children. Int. J. Gen. Med. 11, 91–98. 10.2147/IJGM.S154198 29563828 PMC5849386

[B24] Chung M.-I. Lai M.-H. Yen M.-H. Wu R.-R. Lin C.-N. (1997). Phenolics from *Hypericum geminiflorum* . Phytochemistry 44, 943–947. 10.1016/s0031-9422(96)00644-9

[B25] Emanuel J. Papies J. Galander C. Adler J. M. Heinemann N. Eschke K. (2023). *In vitro* and *in vivo* effects of Pelargonium sidoides DC. root extract EPs® 7630 and selected constituents against SARS-CoV-2 B.1, Delta AY.4/AY.117 and Omicron BA.2. Front. Pharmacol. 14, 1214351. in press. 10.3389/fphar.2023.1214351 37564181 PMC10410074

[B26] Gödecke T. Kaloga M. Kolodziej H. (2005). A phenol glucoside, uncommon coumarins and flavonoides from *Pelargonium sidoides* DC. Z. Naturforsch. B 60, 677–682. 10.1515/znb-2005-0612

[B27] Helmstädter A. (1996). Umckaloabo - late vindication of a secret remedy. Pharm. Hist. 26, 2–4.

[B28] Hutchings A. Scott A. H. Lewis G. Cunningham A. (1996). Zulu medicinal plants. Pietermaritzburg: Natal University Press.

[B29] Kamin W. Funk P. Seifert G. Zimmermann A. Lehmacher W. (2018). EPs 7630 is effective and safe in children under 6 years with acute respiratory tract infections: clinical studies revisited. Curr. Med. Res. Opin. 34, 475–485. 10.1080/03007995.2017.1402754 29119837

[B30] Kayser O. (1997). Phenolische Inhaltsstoffe von Pelargonium sidoides DC. und Untersuchungen zur Wirksamkeit der Umcka-Droge (Pelargonium sidoides DC. und Pelargonium reniforme Curt.). Berlin: PhD Freie Universität.

[B31] Kayser O. Kolodziej H. (1995). Highly oxygenated coumarins from *Pelargonium sidoides* . Phytochemistry 39, 1181–1185. 10.1016/0031-9422(95)00166-5

[B32] Kim C. E. Griffiths W. J. Taylor P. W. (2009). Components derived from Pelargonium stimulate macrophage killing of Mycobacterium species. J. Appl. Microbiol. 106, 1184–1193. 10.1111/j.1365-2672.2008.04085.x 19191950

[B33] Kling H. (1923). Die sieketrooster. Cape Town: Van de Sandt de Villiers.

[B34] Kolodziej H. (2000). Traditionally used *Pelargonium* species: chemistry and biological activity of umckaloabo extracts and their constituents. Curr. Top. Phytochem. 3, 77–93.

[B35] Kolodziej H. (2007). Fascinating metabolic pools of *Pelargonium sidoides* and *Pelargonium reniforme*, traditional and phytomedicinal sources of the herbal medicine Umckaloabo. Phytomedicine 14, 9–17. Suppl 6. 10.1016/j.phymed.2006.11.021 17188477

[B36] Kolodziej H. Kayser O. Tan N. (2002). “Novel coumarin sulphates from *Pelargonium sidoides*: isolation, structure and synthetic approach,” in Natural products in the new millennium: prospects and industrial application. Editors Rauter A. Arauja E. Sales F. Justino J. Santos S. P. (Dordrecht: Springer), 59–64.

[B37] Laidler P. W. (1928). The magic medicine of the hottentots. S. Afr. J. Sci. 25, 433–447.

[B38] Latté K. P. (1999). *Phytochemische und pharmakologische Untersuchungen an Pelargonium reniforme Curt.* PhD. Berlin: Freie Universität.

[B39] Matthys H. Lehmacher W. Zimmermann A. Brandes J. Kamin W. (2016). EPs 7630 in acute respiratory tract infections–a systematic review and meta-analysis of randomized clinical trials. J. Lung. Pulm. Respir. Res. 3, 00068. 10.15406/jlprr.2015.03.00068

[B40] Michaelis M. Doerr H. W. Cinatl J. Jr. (2011). Investigation of the influence of EPs(R) 7630, a herbal drug preparation from *Pelargonium sidoides*, on replication of a broad panel of respiratory viruses. Phytomedicine 18, 384–386. 10.1016/j.phymed.2010.09.008 21036571 PMC7127141

[B41] Misc (1936). “Umckaloaba,” in Royal botanical garden, Kew. Archive File 121317. 1936ff.

[B42] Papies J. Emanuel J. Heinemann N. Kulić Ž. Schroeder S. Tenner B. (2021). Antiviral and immunomodulatory effects of Pelargonium sidoides DC. root extract EPs® 7630 in SARS-CoV-2-infected human lung cells. Front. Pharmacol. 12, 757666. 10.3389/fphar.2021.757666 34759825 PMC8573200

[B43] Pappe L. (1847). A list South African indigenous plants used as remedies by the colonists of the Cape of Good Hope. Cape Town: Pike.

[B44] Pappe L. (1850). Florae capensis medicae prodromus. Cape Town: Robertson, A. S.

[B45] Pereira A. Bester M. Soundy P. Apostolides Z. (2015). Activity-guided isolation and identification of the major antioxidant and anticancer compounds from a commercial *Pelargonium sidoides* tincture. Med. Chem. Res. 24, 3838–3852. 10.1007/s00044-015-1425-6

[B46] Phillips E. P. (1917). A contribution to the flora of the Leribe Plateau and environs. Ann. S. Afr. Mus. 16, 1–379.

[B47] Qasaymeh R. M. Rotondo D. Oosthuizen C. B. Lall N. Seidel V. (2019). Predictive binding affinity of plant-derived natural products towards the protein kinase G enzyme of *Mycobacterium tuberculosis* (Mt PknG). Plants 8, 477. 10.3390/plants8110477 31698813 PMC6918344

[B48] Schapowal A. Dobos G. Cramer H. Ong K. C. Adler M. Zimmermann A. (2019). Treatment of signs and symptoms of the common cold using EPs 7630 - results of a meta-analysis. Heliyon 5, e02904. 10.1016/j.heliyon.2019.e02904 31844762 PMC6888731

[B49] Schildknecht H. (1984). Turgorins—new chemical messengers for plant behaviour. Endeavour 8, 113–117. 10.1016/0160-9327(84)90003-6

[B50] Sechehaye A. (1923). Nouvelle méthode de traitment des diverses formes de la tuberculose par l'umckaloabo. Rev. Med. Suisse Romande 43, 185–192.

[B51] Sechehaye A. (1934) The treatment of pulmonary and surgical tuberculosis with Umckaloabo. Internal medication (Stevens' cure). London: Fraser & Co.

[B52] Seidel V. Taylor P. W. (2004). *In vitro* activity of extracts and constituents of Pelagonium against rapidly growing mycobacteria. Int. J. Antimicrob. Agents 23, 613–619. 10.1016/j.ijantimicag.2003.11.008 15194133

[B53] Seifert G. Brandes-Schramm J. Zimmermann A. Lehmacher W. Kamin W. (2019). Faster recovery and reduced paracetamol use - a meta-analysis of EPs 7630 in children with acute respiratory tract infections. BMC Pediatr. 19, 119. 10.1186/s12887-019-1473-z 31014293 PMC6477747

[B54] Smith A. (1895). A contribution to the South African materia medica. 3. Cape Town: J. C. Juta & Co.

[B55] Smith C. A. (1966). Common names of South African plants. Pretoria: Department of Agricultural Technical Services.

[B56] Stander M. A. Brendler T. Redelinghuys H. Van Wyk B. E. (2019). The commercial history of Cape herbal teas and the analysis of phenolic compounds in historic teas from a depository of 1933. J. Food Compos. Anal. 76, 66–73. 10.1016/j.jfca.2018.11.001

[B57] Stander M. A. Van Wyk B.-E. Taylor M. J. C. Long H. S. (2017). Analysis of phenolic compounds in rooibos tea (*Aspalathus linearis*) with a comparison of flavonoid-based compounds in natural populations of plants from different regions. J. Agric. Food Chem. 65, 10270–10281. 10.1021/acs.jafc.7b03942 29063755

[B58] Teles Y. C. F. Souza M. S. R. Souza M. F. V. (2018). Sulphated flavonoids: biosynthesis, structures, and biological activities. Molecules 23, 480. 10.3390/molecules23020480 29473839 PMC6017314

[B59] Timmer A. Gunther J. Motschall E. Rucker G. Antes G. Kern W. V. (2013). *Pelargonium sidoides* extract for treating acute respiratory tract infections. Cochrane Database Syst. Rev. CD006323. 10.1002/14651858.CD006323.pub3 PMC11835051 24146345

[B60] Viljoen A. M. Zhao J. Sandasi M. Chen W. Khan I. A. (2015). Phytochemical distinction between *Pelargonium sidoides* (“Umckaloabo”) and *P. reniforme* through 1H-NMR and UHPLC–MS metabolomic profiling. Metabolomics 11, 594–602. 10.1007/s11306-014-0722-2

[B61] Watt J. M. Breyer-Brandwijk M. G. (1962). The medicinal and poisonous plants of southern and eastern Africa. 2. London: Livingstone.

