# Supplementary material for: Stevens’ Cure (Umckaloabo)—the vindication of a patent medicine
Source: Front Pharmacol. 2024 Jan 3;14:1294997. doi: 10.3389/fphar.2023.1294997 (PMC10791834; doi:10.3389/fphar.2023.1294997)
Supplement: Supplementary file 1 [file DataSheet1.pdf]

Supplementary Material

SUPPLEMENTARY TABLE 1: Concentrations of umckalin and umckalin sulfate in the historic and commercial samples.

Quantify Compound Summary Report

Compound 1: umckalin

	Sample Text	Conc. mg/Kg
2	*Pelargonium reniforme* radix Batch 20094 Parceval 15/07/2020	N.D.
3	*Pelargonium reniforme* radix Batch 20095 Parceval 15/07/2020	N.D.
4	*Pelargonium sidoides* radix Batch 20090 Parceval 15/07/2020	186.4
5	*Pelargonium sidoides* radix Batch 20091 Parceval 15/07/2020	83.2
6	*Pelargonium sidoides* radix Batch 20092 Parceval 15/07/2020	173.8
7	EBC 45821	363.2
8	EBC 77377	737.7
9	EBC 45819	576.1
11	*Pelargonium reniforme* radix sample a Batch 2023 Parceval 17/04/2023	N.D.
12	*Pelargonium reniforme* radix sample b Batch 2023 Parceval 17/04/2023	N.D.
13	*Pelargonium reniforme* radix sample c Batch 2023 Parceval 17/04/2023	N.D.

Compound 2: Umckalin sulfate

	Sample Text	Conc. mg/Kg
2	*Pelargonium reniforme* radix Batch 20094 Parceval 15/07/2020	N.D.
3	*Pelargonium reniforme* radix Batch 20095 Parceval 15/07/2020	N.D.
4	*Pelargonium sidoides* radix Batch 20090 Parceval 15/07/2020	1521.2
5	*Pelargonium sidoides* radix Batch 20091 Parceval 15/07/2020	840.5
6	*Pelargonium sidoides* radix Batch 20092 Parceval 15/07/2020	1134.1
7	EBC 45821	3.4
8	EBC 77377	85.4
9	EBC 45819	62.9
11	*Pelargonium reniforme* radix sample a Batch 2023 Parceval 17/04/2023	N.D.
12	*Pelargonium reniforme* radix sample b Batch 2023 Parceval 17/04/2023	N.D.
13	*Pelargonium reniforme* radix sample c Batch 2023 Parceval 17/04/2023	N.D.

N.D.- not detected

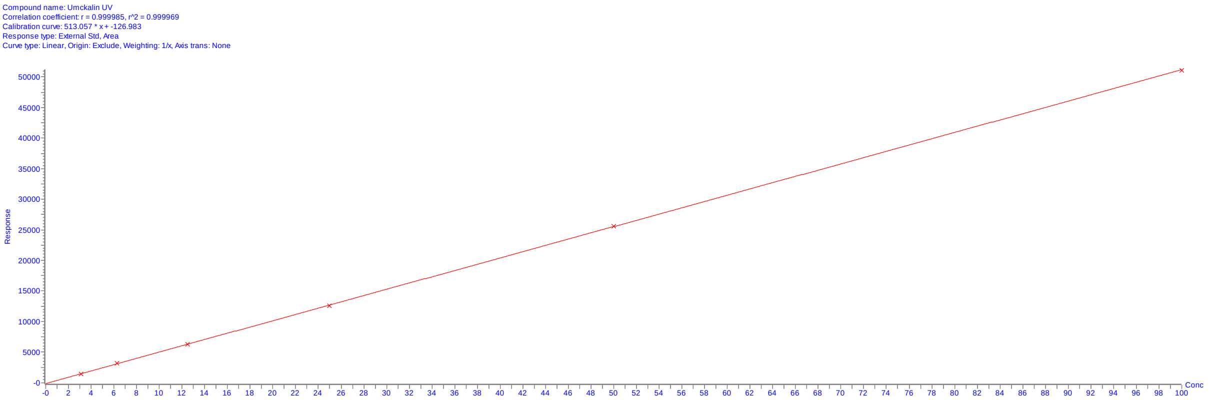

SUPPLEMENTARY FIGURE 1: Calibration curve of the umckalin standard. Detection limit for umckalin: 0.2 mg/kg, determined as the lowest concentration detectable at a signal to noise of 3 or higher. No umckalin or umckalin sulphate was detected in the *P reniforme* samples. No umckalin sulphate standard was available, the samples were hydrolysed to convert it to umckalin by the addition of 0.6 HCl and analysed again against the umckalin standard.

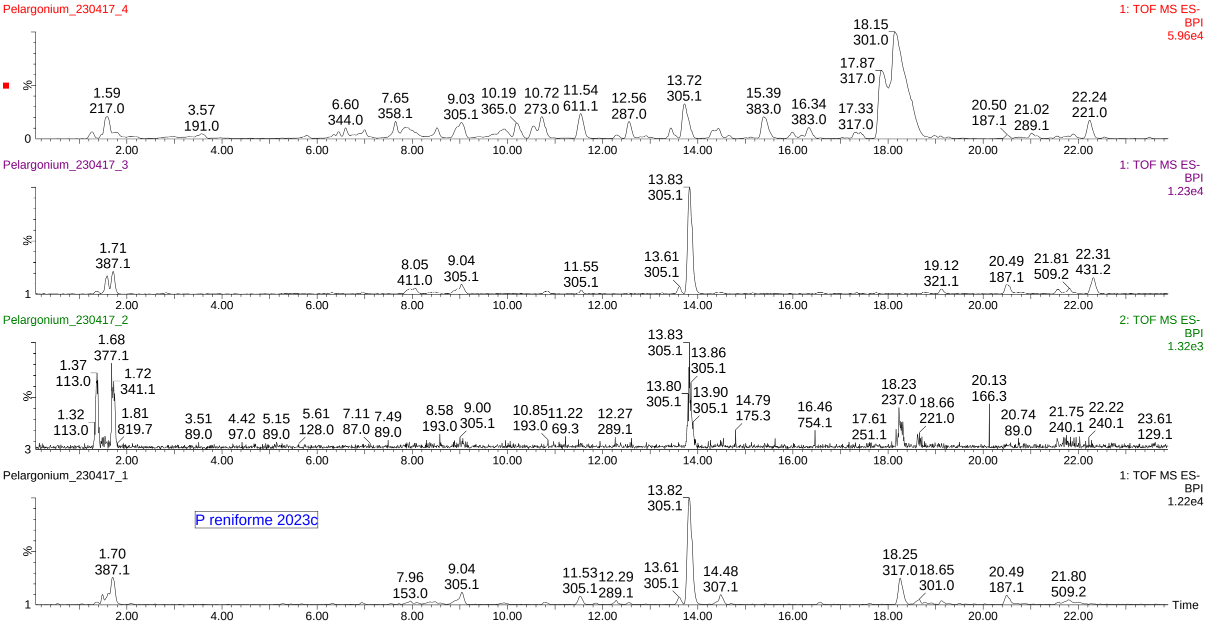

SUPPLEMENTARY FIGURE 2: Total ion chromatograms of 3 *P. reniforme* extracts from 2023 with a *P sidoides* extract on top. The large peak at 18 min, *m/z* 301 in the chromatogram on top is the umckalin sulfate and it clearly not present in the 3 chromatograms below it.

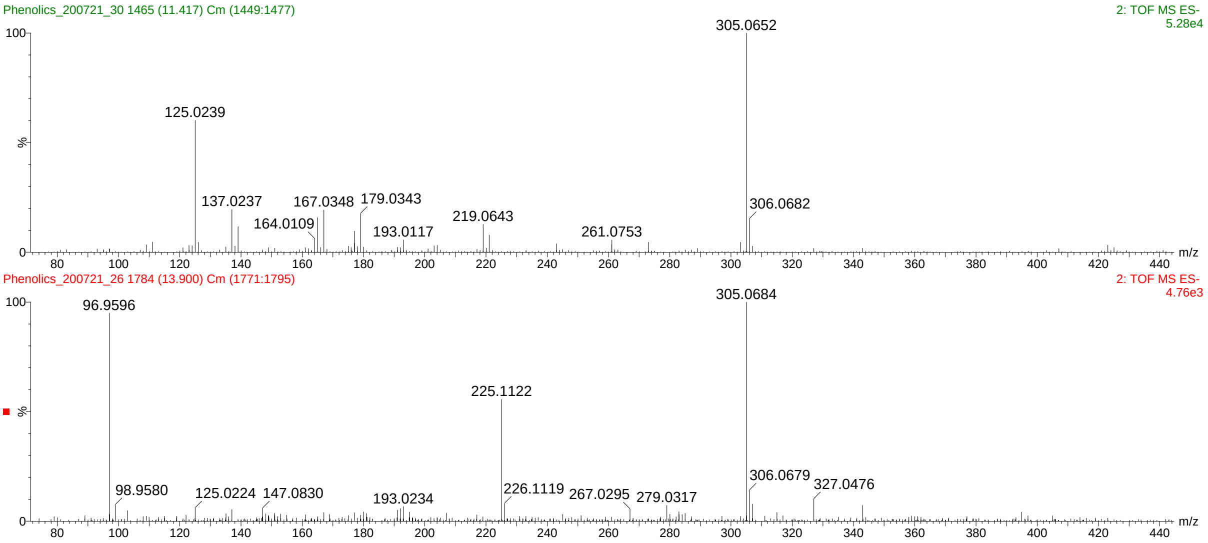

SUPPLEMENTARY FIGURE 3: MS^E fragmentation spectra of gallocatechin and unknown sulphated flavonoid below (refer to TABLE 1 in main document)

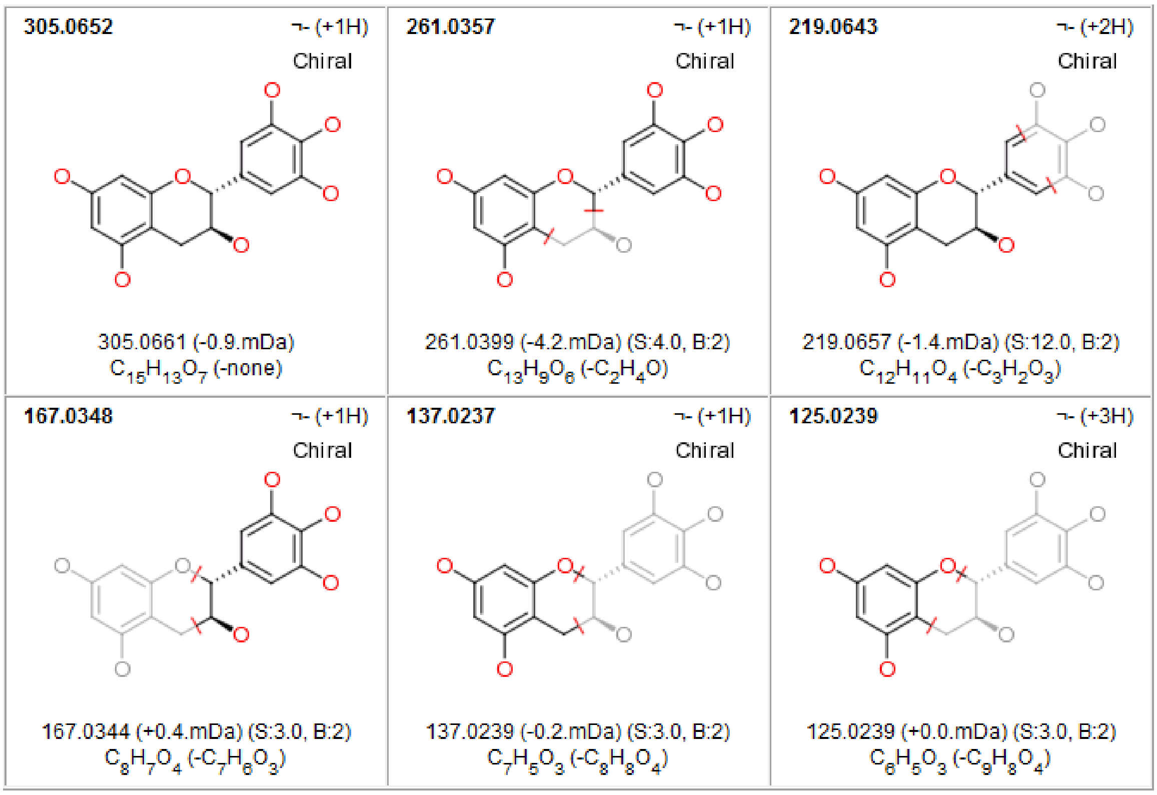

SUPPLEMENTARY FIGURE 4: Predicted fragmentation for gallocatechin, using the structure and fragmentation data in the Massfragment application of Masslynx 4.1 (Waters, US)

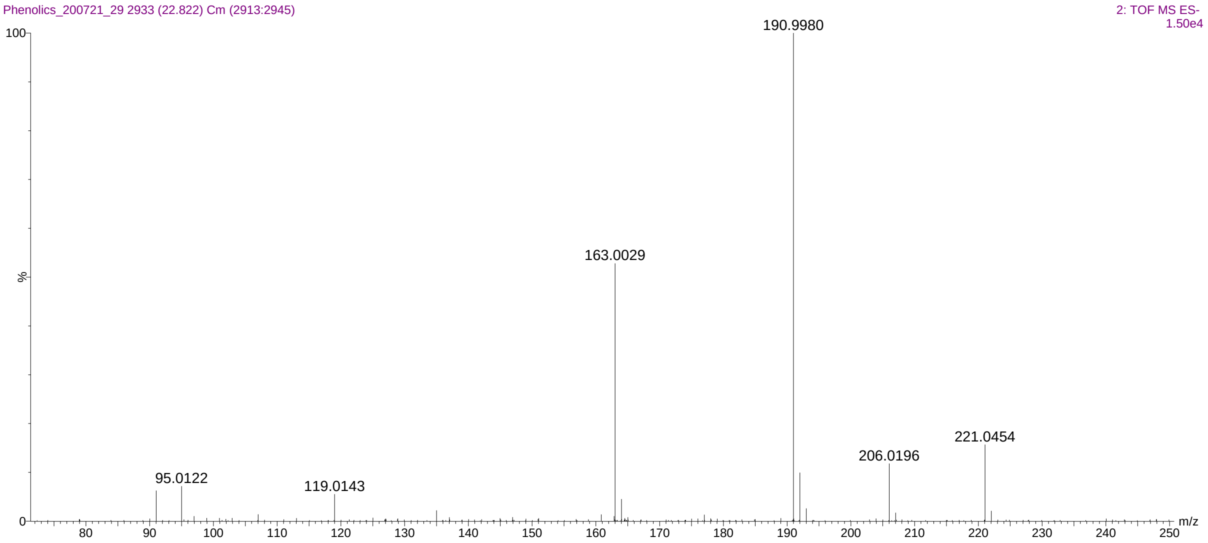

SUPPLEMENTARY FIGURE 5: MS^E fragmentation spectra of umckalin (refer to TABLE 1 in main document)

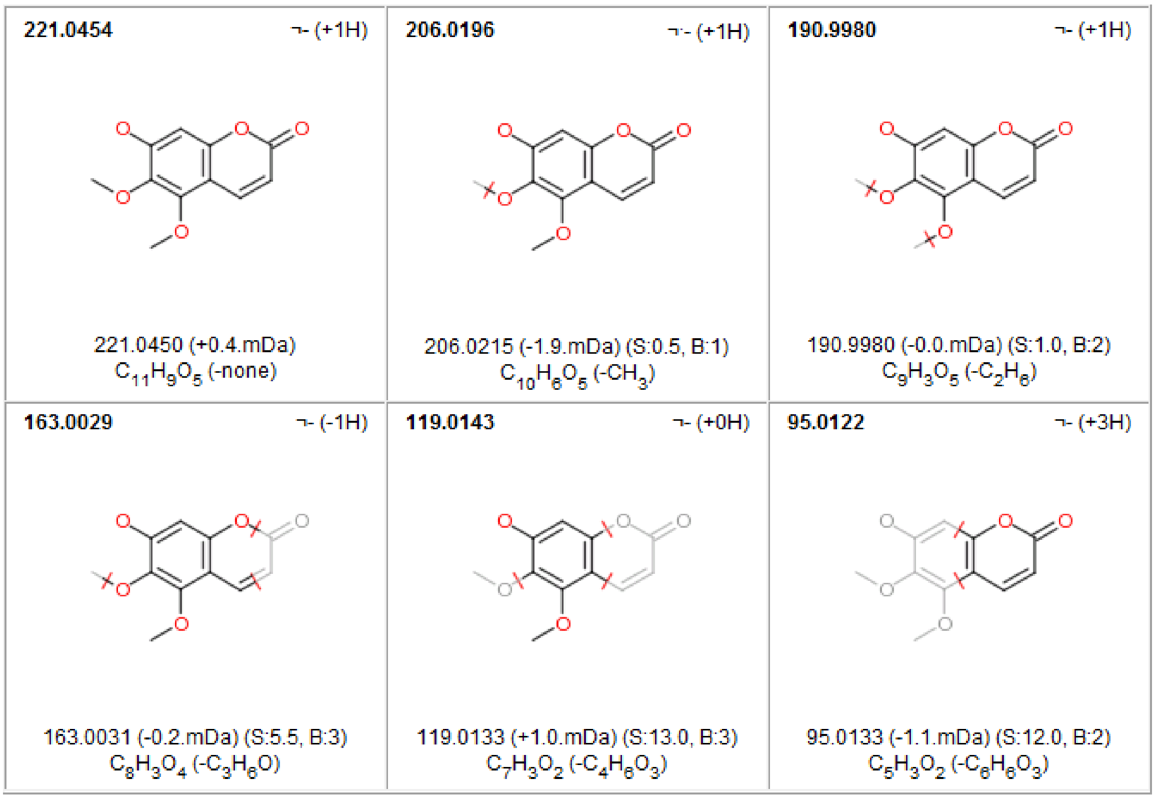

SUPPLEMENTARY FIGURE 6: Predicted fragmentation for umckalin, using the structure and fragmentation data in the Massfragment application of Masslynx 4.1 (Waters, US)

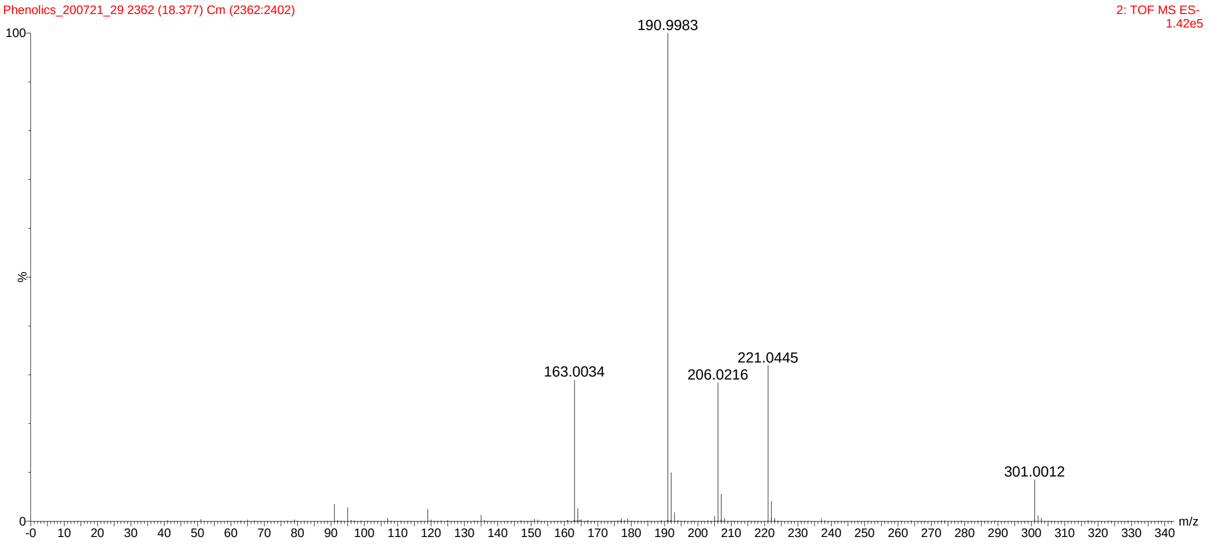

SUPPLEMENTARY FIGURE 7: MS^E fragmentation spectra of umckalin sulphate (refer to TABLE 1 in main document)

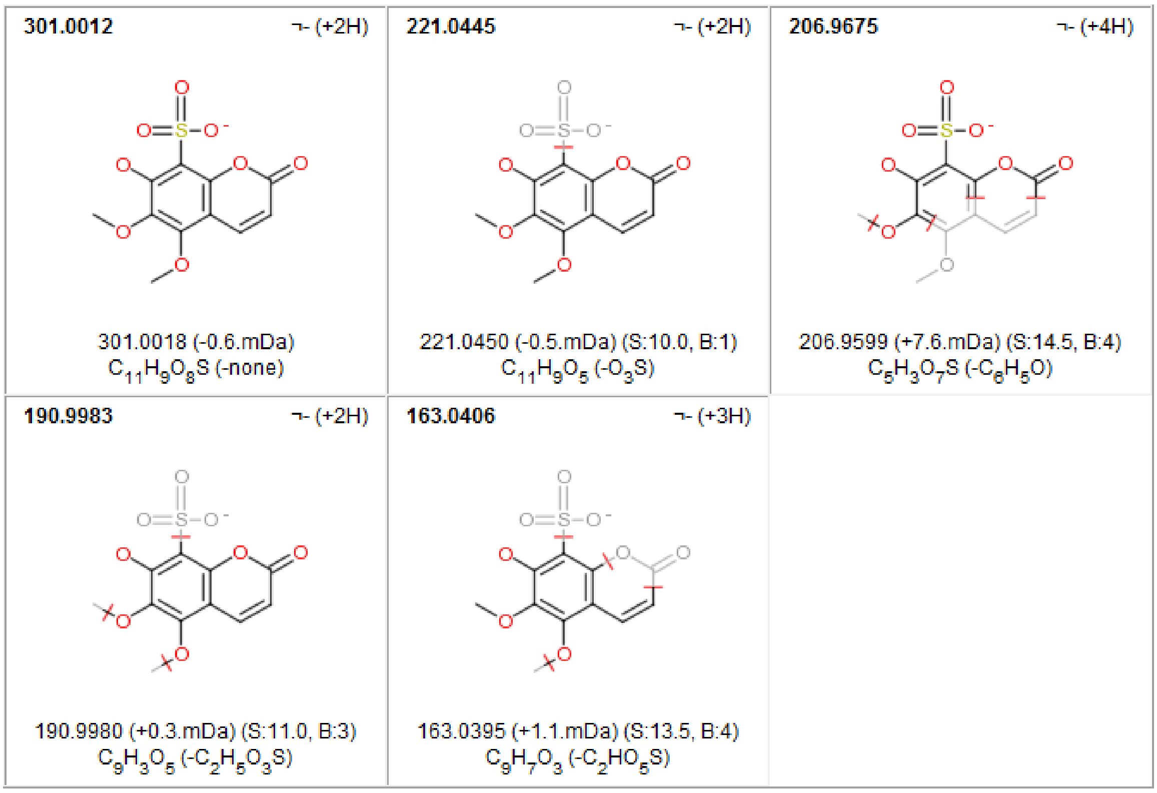

SUPPLEMENTARY FIGURE 8: Predicted fragmentation for umckalin sulphate, using the structure and fragmentation data in the Massfragment application of Masslynx 4.1 (Waters, US)
